# Efficacy of mouthwash on reducing salivary SARS-CoV-2 viral load and clinical symptoms: a systematic review and meta-analysis

**DOI:** 10.1186/s12879-023-08669-z

**Published:** 2023-10-11

**Authors:** Mingrui Zhang, Nan Meng, Hong Duo, Yuanbo Yang, Qing Dong, Jianqi Gu

**Affiliations:** 1 North China University of Science and Technology, No. 21, Bohai Avenue, Caofeidian New Town, Tangshan City, 063000 Hebei China; 2 Department of Critical Care Medicine, Zhongnan Hospital of Wuhan University, The Second Clinical College of Wuhan University, Wuhan, 430071 China; 3 Department of Stomatology, Tangshan Workers Hospital, Tangshan, 063000 Hebei China; 4 Department of Stomatology, HeBei General Hospital, Shijiazhuang, 050000 Hebei China

**Keywords:** COVID-19, Mouthwash, Virus transmission, Randomized controlled trials, Povidone-iodine

## Abstract

**Background:**

COVID-19 has been a public health emergency of international concern (PHEIC) for a lengthy period of time. The novel coronavirus is primarily spread via aerosols at a short distance, with infected individuals releasing large amounts of aerosols when speaking and coughing. However, there is an open question regarding whether mouthwash could effectively reduce virus transmission during the COVID-19 pandemic and support the prevention of infection among medical workers.

**Methods:**

Cochrane Library, PubMed, Web of Science, and Embase databases were systematically searched from the inception of each database to January 12, 2023 for currently available randomized clinical trials (RCTs) on the effect of mouthwash on novel coronavirus load in the oral cavity in COVID-19 patients. The treatment group received mouthwash for rinsing the mouth, while the control group received a placebo or distilled water for COVID-19 patients. The primary outcomes were CT value and viral load. Odds ratios (ORs) were estimated using a random-effects model. Subgroup and sensitivity analyses were performed to minimize the bias and the impact of heterogeneity.

**Results:**

Thirteen RCTs were included. Seven studies reported the intervention effect of mouthwash on the CT value of novel coronavirus. The analysis results showed that the mouthwash group had a positive impact on the CT value of novel coronavirus [ SMD = 0.35, 95% CI (0.21, 0.50)] compared with the control group. In addition, subgroup analysis showed a significant positive effect of mouthwash on CT values in the treatment group compared with the control group, with chlorhexidine (CHX) [SMD = 0.33, 95% CI (0.10, 0.56)], povidone-iodine (PVP-I) [SMD = 0.61, 95% CI (0.23, 0.99)], or hydrogen peroxide (HP) [SMD = 1.04, 95% CI (0.30, 1.78)] as an ingredient of the mouthwash. Six studies reported the intervention effect of mouthwash on the viral load, 263 cases in the treatment group and 164 cases in the control group. The analysis results showed that there was no statistical difference between the mouthwash group and the control group in the viral load of novel coronavirus [SMD = -0.06, 95% CI (-0.18, 0.05)]. In the subgroup analysis by measurement time, there were statistically significant differences between the mouthwash and control groups for CT values [SMD = 0.52, 95% CI (0.31, 0.72)] and viral load [SMD =  − 0.32, 95% CI (− 0.56, − 0.07)] within 30 min of gargling.

**Conclusions:**

In summary, mouthwash has some efficacy in reducing the viral load of novel coronavirus, especially within 30 min after rinsing the mouth. Mouthwash containing CHX, PVP-I and HP all had significant positive effects on CT values, and PVP-I-containing mouthwash may be a promising option to control novel coronavirus infections and relieve virus-related symptoms. However, studies on the dose and frequency of use of mouthwash for infection control are still lacking, which may limit the clinical application of mouthwash.

**Trial registration:**

Protocol registration: The protocol was registered at PROSPERO (CRD42023401961).

**Supplementary Information:**

The online version contains supplementary material available at 10.1186/s12879-023-08669-z.

## Introduction

Since December 31, 2019, the outbreak of Coronavirus Disease 2019 (COVID-19) caused by the 2019 novel coronavirus ((2019-nCoV)) has seriously threatened public health [1]. As of May 31, 2022, the Institute for Health Metrics and Evaluation (IHME) reported 6.9 million deaths due to COVID-19, with an estimated 17.2 million deaths [2]. At the same time, the increasing prevalence of coronavirus reinfection and long-term COVID-19 have weakened millions of people, and the number continues to increase [3].

Despite efforts to contain the virus, it continues to mutate, and as recently as January 30, 2023, the Director-General of the World Health Organization announced that COVID-19 had been a public health emergency of international concern (PHEIC) over a lengthy period of time [4]. Therefore, it is crucial to prepare for potential future outbreaks with effective public infection control measures.

The novel coronavirus is primarily spread via aerosols at a short distance, with infected individuals releasing large amounts of aerosols when speaking and coughing [2]. The microorganism found in dental bioaerosols are mainly attributed to patients' nasopharyngeal secretions, saliva, blood, and dental unit waterlines [5]. With this in mind, the US Occupational Safety and Health Administration (OSHA 2020) listed the dental department as one of the occupations with the highest risk of SARS-CoV-2 transmission, indicating that healthcare professionals in the department of stomatology and otolaryngology should take measures to prevent infection when treating patients with the novel coronavirus.

Previously, preprocedural mouth rinsing has been applied before routine oral treatment as an important method for healthcare professionals to reduce contamination. Chlorhexidine (CHX) is recommended as the gold standard of mouthrinse for chemical control of supragingival biofilm. Among antiviral molecules contained in mouthwashes, hydrogen peroxide (HP), β-cyclodextrin, flavonoids, essential oils, cetylpyridinium chloride (CPC) or povidone-iodine (PVP-I) could be useful in the fight against SARS-CoV-2 [6–8]. In the early stage of the COVID-19 pandemic, many international guidelines and articles recommended the use of mouthwashes containing hydrogen peroxide (H2O2) and povidone-iodine (PVP-I) against SARS-CoV-2 [9, 10]. CPC is recommended for rinsing the mouth by the National Dental Center of Singapore [11]. To date, the conclusions of studies on the effect of mouthwashes on SARS-CoV-2 viral load remain inconsistent.

The aim of this study was to investigate whether mouthwash could effectively reduce virus transmission during the COVID-19 pandemic and provide evidence to support the prevention of infection among medical workers, through the collection of randomized controlled trial studies (RCTs).

## Materials and methods

### Protocol and registration

This meta-analysis is performed based on Cochrane Handbook for the Systematic Review of Interventions (for details, see http://training.cochrane.org/handbook) and the Preferred Reporting Items for Systematic Review and Meta-Analyses [12]. This study protocol was approved in PROSPERO (registration number: CRD42023401961).

### Eligibility criteria

#### Inclusion criteria

Inclusion criteria followed the PICOS strategy.

P (Population): The study population consisted of adult patients who were diagnosed with COVID-19 and not allergic to mouthwash ingredients.

I (Intervention): The interventions were performed with an experimental antiseptic mouthwash.

C (Comparison): The comparisons included placebo or distilled water.

O (Outcome): The outcomes assessed were cycle threshold (CT) values of polymerase chain reaction (PCR) assay or values of viral load as copies/ml.

S (Study design): The study design belonged to randomized controlled trials (RCTs).

#### Exclusion criteria

Exclusion criteria followed the PICOS strategy.

P (Population): Studies in which the study population had other diseases were excluded.

I (Intervention): Studies in which mouthwash was not used as the interventions or used in conjunction with other treatments were excluded.

C (Comparison): Studies in which the comparison used other mouthwashes or mouthwash duration as controls were excluded.

O (Outcome): Studies that did not assess outcomes of interest or with incomplete data were excluded.

S (Study design): Non-randomized controlled trials, reviews, case reports, animal experiments, in vitro studies, and observational study designs were excluded. Articles without full texts were also excluded.

### Search strategy

The Cochrane Library, PubMed, Web of Science, and Embase databases were searched for studies on the effect of mouthwash on novel coronavirus load in oral cavity. The search period was from inception of each database to January 12, 2023. The search strategy of a combination of subject terms and free words was used, and the search terms included "mouthwash", "mouthrinse", "COVID-19" and "SARS-CoV-2". Specific search strategies are presented in Table S1.

### Literature screening and data extraction

Two researchers searched the literature in strict accordance with the inclusion and exclusion criteria, and Endnote X9 was used to manage all literature. The retrieved literature was imported into Endnote X9. After duplicate publications were excluded, the preliminarily eligible studies were screened out based on titles or abstracts, and their full texts were downloaded. After the full texts were read, the original studies that met this systematic review were screened out. Literature data were extracted and cross-checked, and units of measurement were unified. Disagreements, if any, were resolved by discussion with a third researcher. The extracted data mainly included the first author, publication year, country, type of study, sample size and age distribution of the treatment group and control group, mouthwash ingredients, follow-up time, and outcome measures.

### Risk of bias assessment for the included studies

The assessment work was performed by two researchers separately and the results were cross-checked. Cochrane Handbook for Systematic Reviews of Interventions version 6.3, Chapter 8: To assess the risk of bias in a randomized trial, the Cochrane risk-of-bias tool for randomized trials (RoB 2) was adopted for quality evaluation of the included studies, and the results were cross-validated. The assessment items include seven aspects: generation of random sequences (selection bias), allocation concealment (selection bias), blinding of investigators and subjects (implementation bias), blinding evaluation for study outcomes (measurement bias), integrity of outcome data (follow-up bias), selective reporting of study results (reporting bias), and other sources (other biases). The Review Manager (RevMan) Version 5.4, and [Computer program. The Cochrane Collaboration, 2020.] software were used to draw the risk of bias graph and summary figure.

### Statistical methods

Stata 15.0 software was used for statistical analysis of the included studies, including heterogeneity test, publication bias analysis, and sensitivity analysis. Continuous variables were pooled using standard mean difference (SMD) and the 95% confidence interval (CI) was calculated, while binary variables were pooled using relative risk (RR) and 95% confidence interval (CI) was calculated. Q statistic and I^2^ test were used to evaluate heterogeneity. *P* > 0.1 and I^2^ ≤ 50% indicated acceptable heterogeneity among studies, and the fixed effects model was adopted for meta-analysis; *P* ≤ 0.1 or I^2^ > 50% indicated greater heterogeneity among studies, and the random effects model was selected for meta-analysis. The "metabias" command was used to detect publication bias of the included studies, and for all results, *P* < 0.05 was considered statistically significant. When there is a publication bias exist, a funnel plot was further analyzed using the trim-and-fill method by entering the code of 'metatrim _ES _selogES, funnel'.

## Results

### Literature search results

**Fig. 1:**
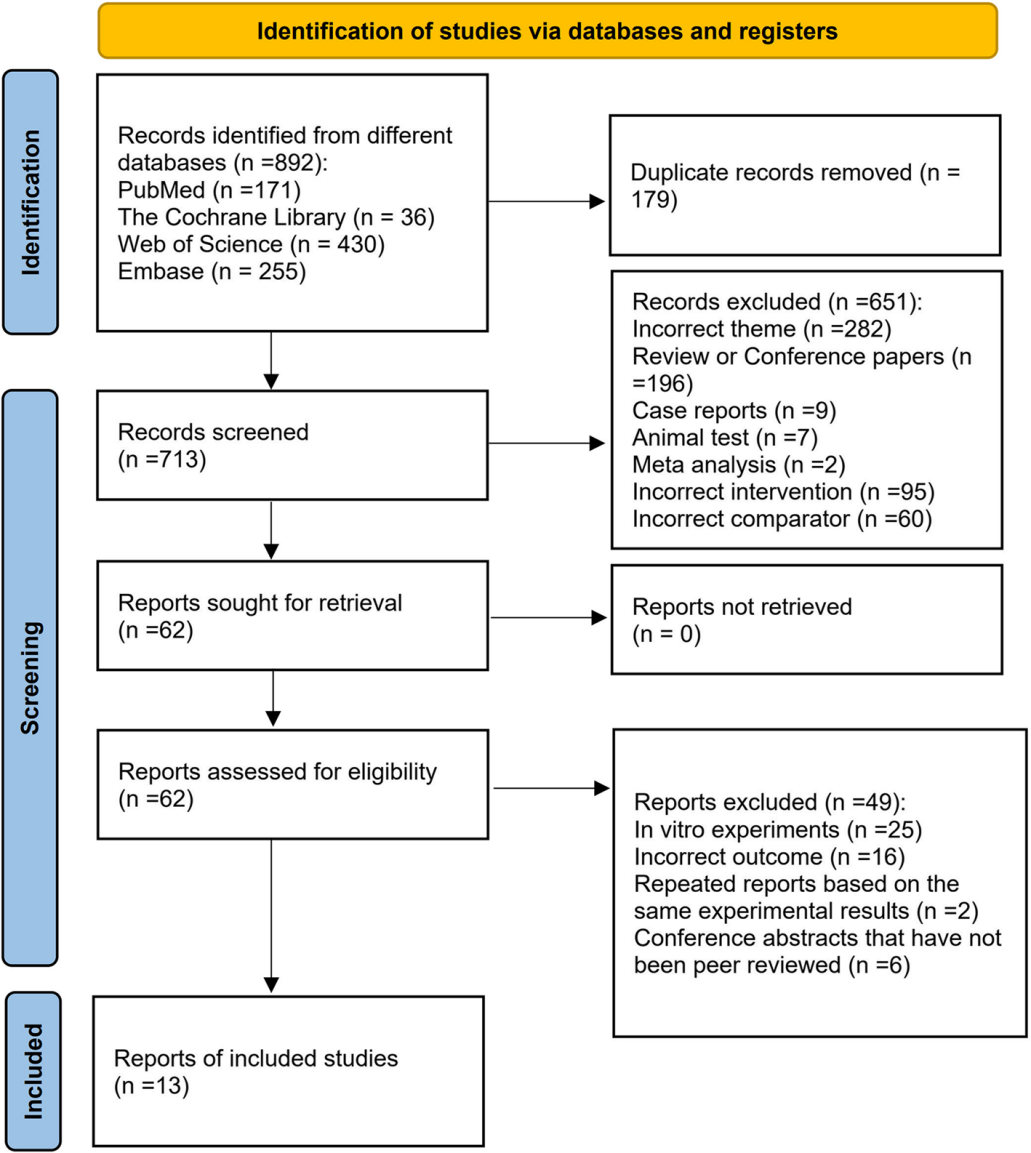
Flow diagram

A total of 892 publications were obtained. The retrieved studies were imported into EndNote X9, and 179 duplicated articles were eliminated. A total of 651 irrelevant articles were eliminated by reading the titles and abstracts, 49 articles that did not meet the criteria were eliminated by reading the full texts, and 13 articles were finally included in the present study [11, 13–24]. Literature screening process and results are shown in Fig. 1.

### Basic characteristics of the included literature

Thirteen articles were included [11, 13–24], involving 832 subjects, with 523 in the treatment group and 309 in the control group. All literature reporting related intervention indicators were English publications. The basic characteristics of the included literature are shown in Table 1.

**Table 1:** Characteristics of the 13 RCTs

Author Publication Year	Country	Type of study	Sample Size		Age	Mouthwash ingredients	Follow-up duration	Outcome Measures
			Treatment Group	Control Group				
Zuhair S. Natto 2022 [21]	Saudi Arabia	RCT	30	15	37.3 ± 13.2	CHX; PVP-I;	up to 5 min	①
Fernanda de Paula Eduardo 2021 [17]	Brazil	RCT	34	9	54.67 ± 12.46	CPC + Zn HP CHX HP + CHX	60 in	①
Rosa Tarragó-Gil 2023 [24]	Spain	RCT	39	40	48.6 ± 15.5	CPC	2 h	①
Rola Elzein 2021 [18]	Lebanon	RCT	52	9	45.3 ± 16.7	CHX PVP-I	Post-wash	①
Chaminda J. Seneviratne 2021 [11]	Singapore	RCT	14	2	39.97 ± 9.67	PVP-I CHX CPC	6 h	①
Sema Nur Sevinç Gül 2022 [23]	Turkey	RCT	41	20	51.67 ± 18.81	HClO, PVP-I	Post-wash	①
Denis Damião Costa 2022 [16]	Brazil	RCT	50	50	39.49 ± 12.69	CHX	60 min	①
Florence Carrouel 2021 [15]	France	RCT	88	88	43.06 ± 5.56	β-cyclodextrin and citrox	7 days	②
Manar M. Alzahrani 2023 [14]	Saudi Arabia	RCT	37	8	37.18 ± 10.93	PVP-I CPC H2O2 HOCl	60 min	②
A. Alemany 2022 [13]	Spain	RCT	40	40	45.95 ± 13.47	CPC	3 h	②
Toni Luise Meister 2022 [20]	Germany	RCT	18	6	29.13 ± 10.87	BAC	30 min	②
Álvaro Sánchez Barrueco 2022 [22]	Spain	RCT	34	10	61.71 ± 12.61	PVP-I H2O2 CPC CHX	7 days	②
Ferrer, M. D 2021 [19]	Korea	RCT	46	12	54.5 ± 17	PVP-I H2O2 CPC CHX	2 h	②

① CT value

② Viral load

### Risk of bias assessment

**Fig. 2:**
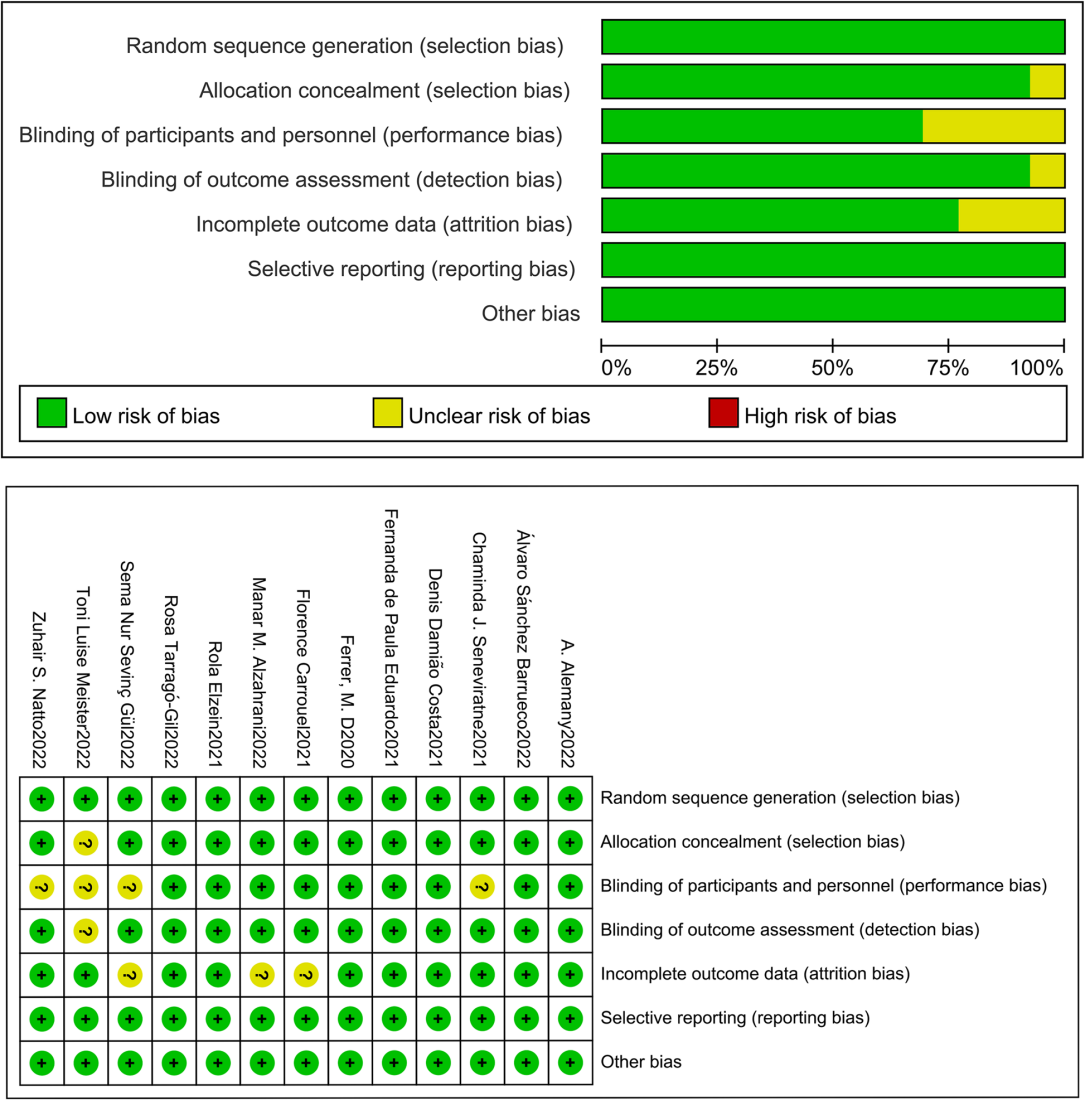
Quality analysis

The Cochrane risk-of-bias tool was used to assess the quality of the 13 included RCTs, which were at low or unknown risk in each scoring item, including the generation of random sequence, blinding, allocation concealment, integrity of outcome data, and selective reporting of study results. Assessment results are shown in Fig. 2

### Meta-analysis results

#### Meta-analysis results of CT value

**Fig. 3:**
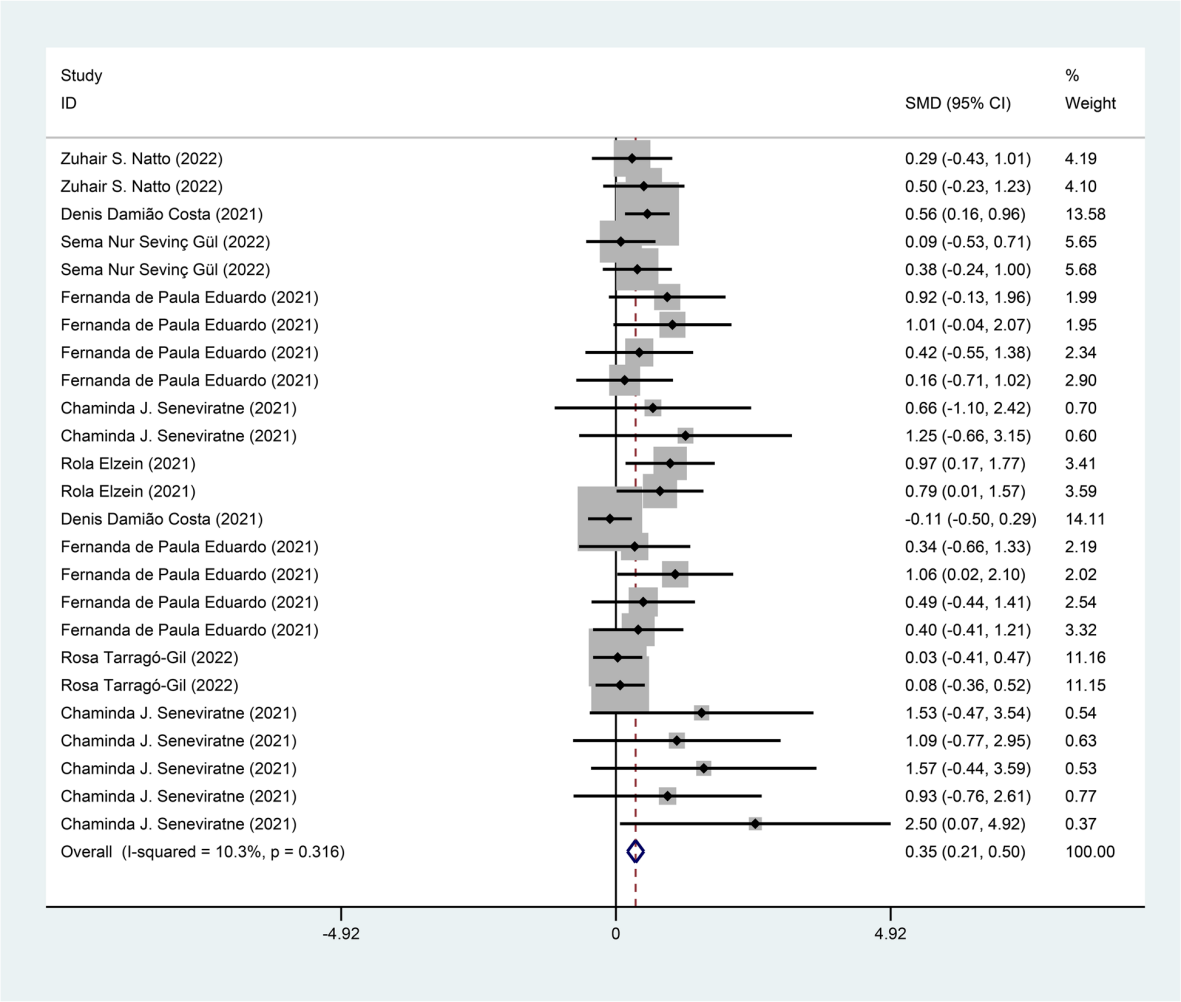
Forest plot of CT values

Seven studies [11, 16–18, 21, 23, 24] reported the intervention effect of mouthwash on the CT value of novel coronavirus, with 260 cases in the treatment group and 145 cases in the control group. The fixed effects model (I^2^ = 10.3%, *P* = 0.316) was used to pool the effect size, and the analysis results showed a positive effect in terms of CT value of novel coronavirus [SMD = 0.35, 95% CI (0.21, 0.50)] in the mouthwash group compared with the control group as shown in Fig. 3.

**Fig. 4:**
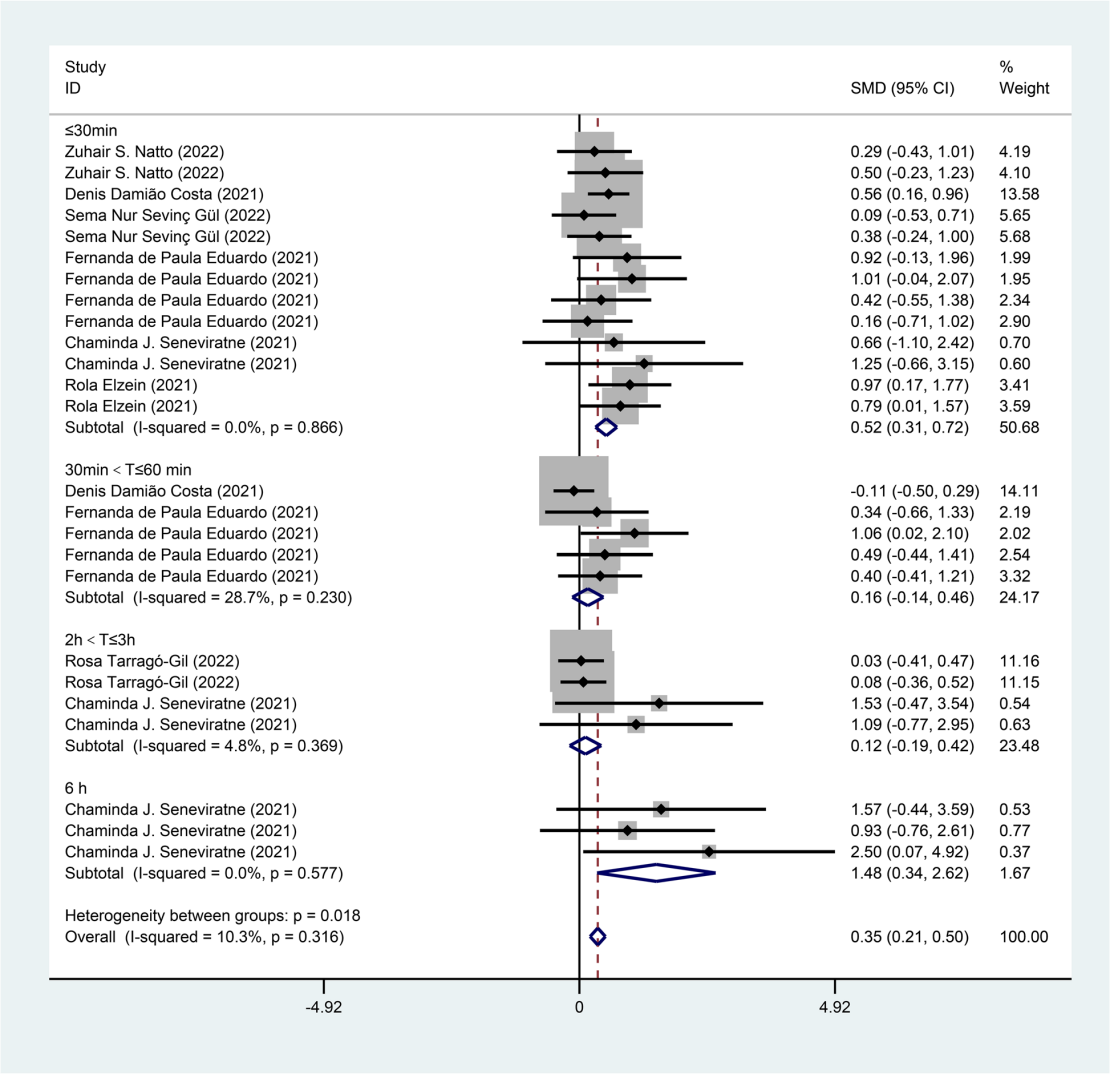
Forest plot of CT values for subgroup analysis by measurement time

Subgroup analyses were performed based on measurement time and different mouthwash ingredients. Subgroup analysis by measurement time revealed that mouthwash showed a significant positive effect on CT values within 30 min [SMD = 0.52, 95% CI (0.31, 0.72)] and after six hours [SMD = 1.48, 95% CI (0.34, 2.62)] compared with the control group, while there was no statistical difference between the treatment group and the control group within the periods of 30 min-60 min [SMD = 0.16, 95% CI (− 0.14, 0.46)] and 2 h–3 h [SMD = 0.12, 95% CI (− 0.19, 0.42)] as shown in Fig. 4.

**Fig. 5:**
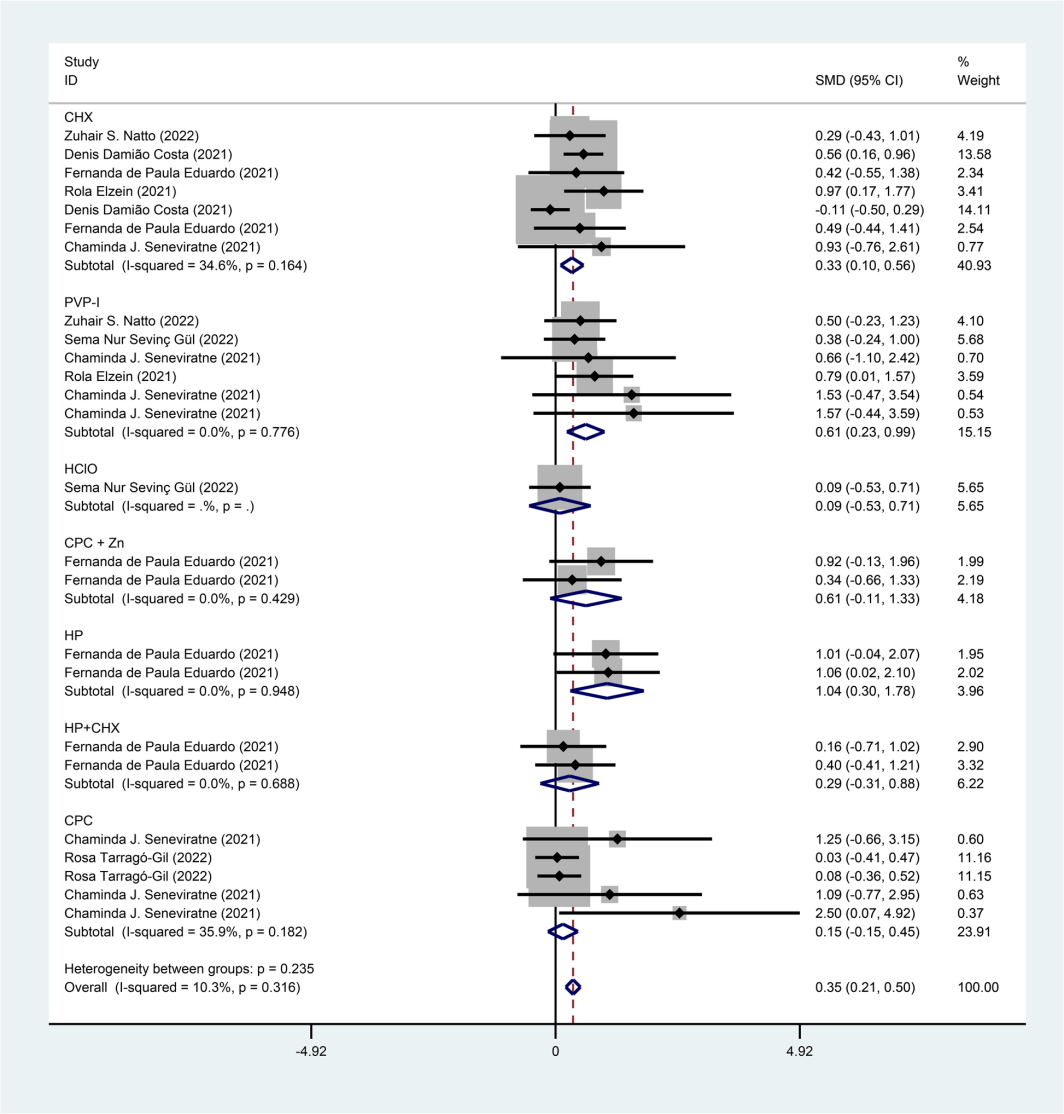
Forest plot of CT values for subgroup analysis by mouthwash ingredients

Subgroup analysis by mouthwash ingredients showed a significant positive effect on CT values in the treatment group compared with the control group with CHX [SMD = 0.33, 95% CI (0.10, 0.56)], PVP-I [SMD = 0.61, 95% CI (0.23, 0.99)], or HP [SMD = 1.04, 95% CI (0.30, 1.78)] as an ingredient of the mouthwash, and indicated no statistically significant difference between the treatment group and the control group with HCIO [SMD = 0.09, 95% CI (-0.53, 0.71)], CPC + Zn [SMD = 0.61, 95% CI (-0.11, 1.39)], HP + CHX [SMD = 0.29, 95% CI (-0.31, 0.88)] and CPC [SMD = 0.15, 95% CI (-0.15, 0.45)] as an ingredient of the mouthwash as shown in Fig. 5.

### Meta-analysis results of viral load

**Fig. 6:**
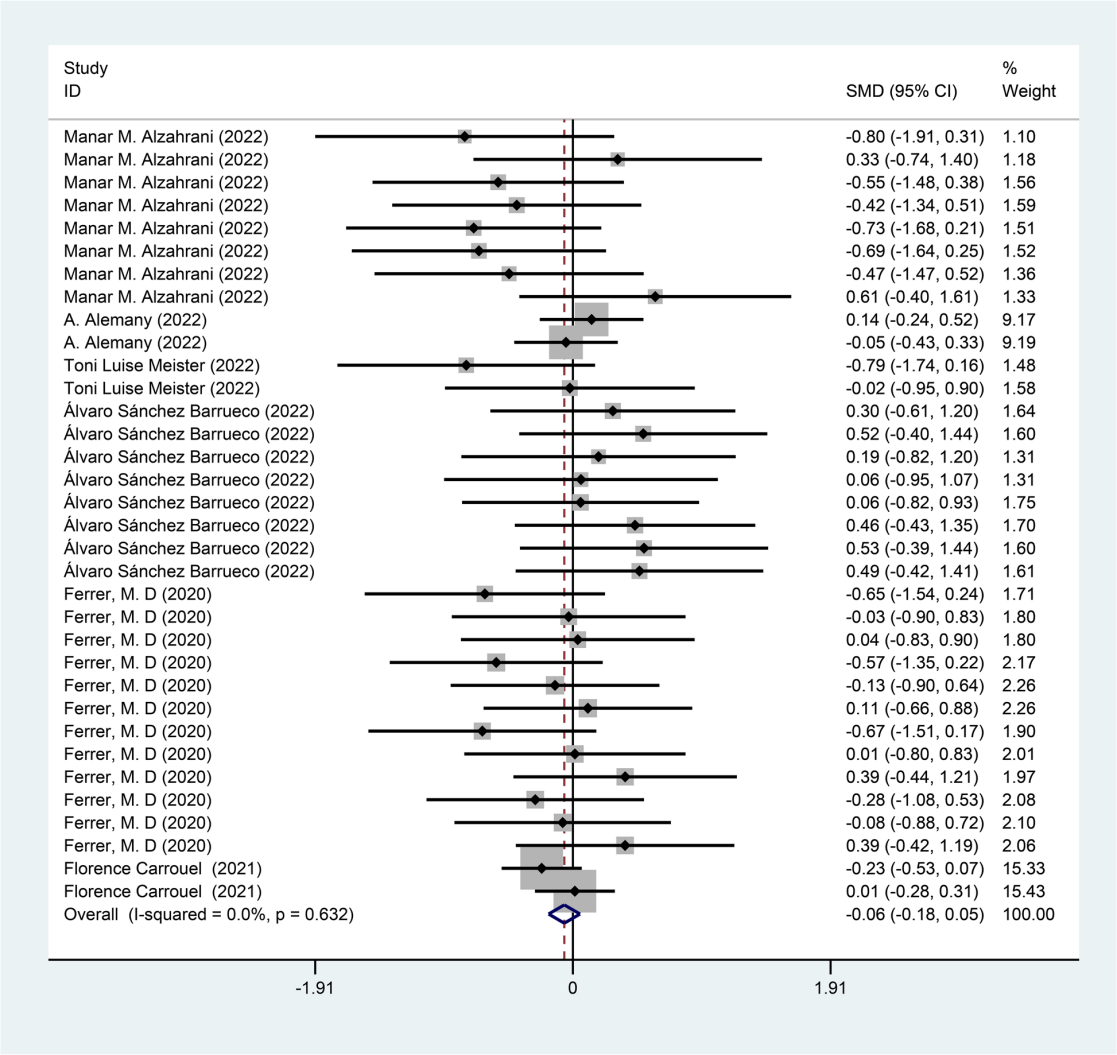
Forest plot of viral loads

Six studies [13–15, 19, 20, 22] reported the intervention effect of mouthwash on viral load, with 263 cases in the treatment group and 164 cases in the control group. The fixed effect model (I^2^ = 0.0%, *P* = 0.632) was used to pool the effect size, and the analysis results showed that there was no statistical difference in viral load value of novel coronavirus between mouthwash group and the control group [SMD = -0.06, 95% CI (-0.18, 0.05)] as shown in Fig. 6. Subgroup analysis were performed based on measurement time and different mouthwash ingredients.

**Fig. 7:**
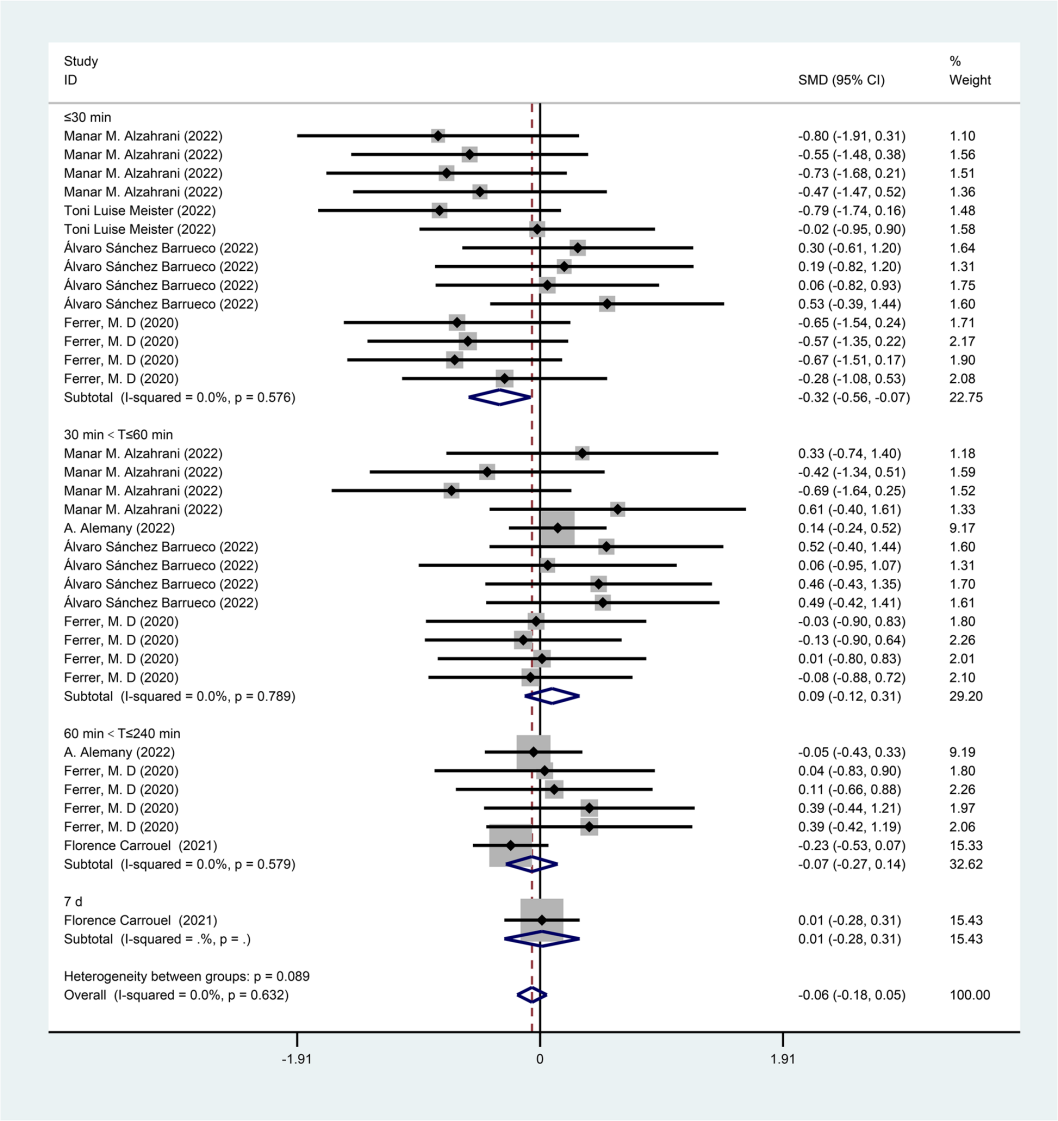
Forest plot of viral loads for subgroup analysis by measurement time

Subgroup analysis by measurement time showed significantly lower viral load values within 30 min [SMD =  − 0.32, 95% CI (− 0.56, − 0.07)] in the mouthwash group compared with the control group, while there was no statistical difference between the treatment group and the control group within the periods of 30 min-60 min [SMD = 0.09, 95% CI (− 0.12, 0.31)], 60 min-240 min [SMD = -0.07, 95% CI (-0.27, 0.14)] and on day seven [SMD = 0.01, 95% CI (− 0.28, 0.31)] as shown in Fig. 7.

**Fig. 8:**
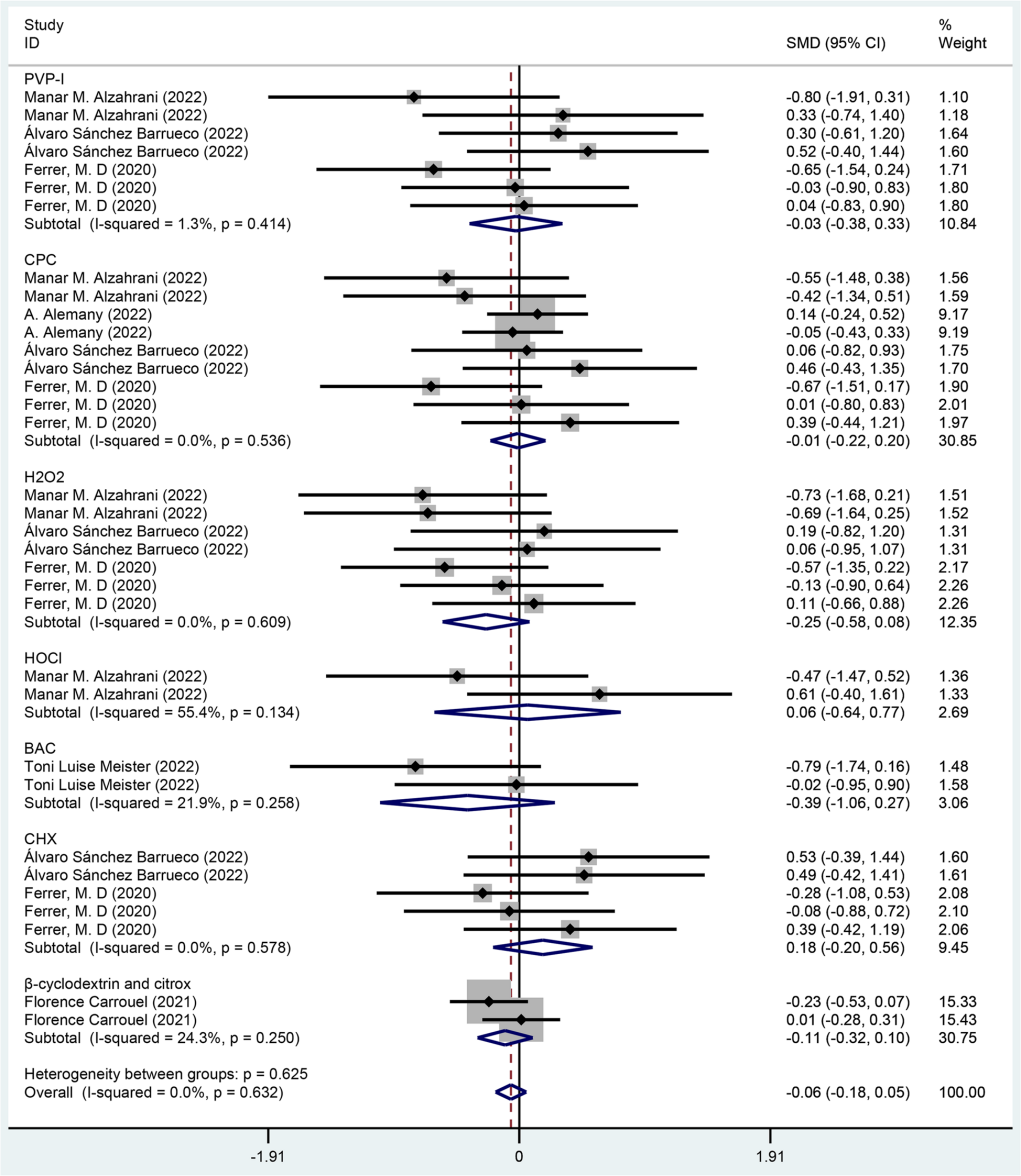
Forest plot of viral loads for subgroup analysis by mouthwash ingredients

Subgroup analysis by mouthwash ingredients showed no statistical differences between treatment group and control group, and the results were as follows: PVP-I [SMD = -0.03, 95% CI (-0.38, 0.33)], CPC [SMD = -0.01, 95% CI (-0.22, 0.20)], H2O2 [SMD = -0.25, 95% CI (-0.58,0.08)], HOCI [SMD = 0.06, 95% CI (-0.64, 0.77)], BAC [SMD = -0.39, 95% CI (-1.06.0.27)], CHX [SMD = 0.18, 95% CI (-0.20, 0.56)], β-cyclodextrin and citrox [SMD = -0.11, 95% CI (-0.32, 0.10)] as shown in Fig. 8.

### Sensitivity analysis and publication bias

There were no sensitivity problems for each of the included indicators (Figure S1, S2). A funnel plot was used to visually display publication bias, and Egger's test was used to analyze the funnel plot of the included studies (Figure S3, S4). A value of *P* > 0.05 in Egger's test indicated no publication bias existed. For the indicators of the included literature in this study, there was bias in the CT values (*p* = 0.000), and no publication bias in the viral loads (Table 2). The indicators with publication bias were further analyzed with the trim-and-fill method, and the funnel plot became symmetrical after adding 10 studies to the model (Figure S5), with a pooled effect size of 0.244 (0.106, 0.383) (Table 3).

**Table 2:** Publication bias of the13RCTs

	**Items**	**Effect size**	**Standard error**	**95%CI**	**t value**	***p***
CT value	slope	-0.192	0.121	-0.443, 0.059	-1.59	0.126
	bias	1.633	0.323	0.966, 2.301	5.06	0.000
Viral Load	slope	-0.042	0.134	-0.316, 0.232	-0.31	0.756
	bias	-0.069	0.389	-0.862, 0.725	-0.18	0.861

**Table 3:** Results of trim-and-fill method

Outcome Measures	Method	Phase	Pooled Est	95% CI	p	No. of studies
CT value	Fixed	Before	0.353	0.206 0.500	0.000	25
		After	0.244	0.106 0.383	0.001	35

## Discussion

The oral cavity is the second most complex microbiota of the human body, and most of these microorganisms are inseparable from human health. In 2019, 2019-nCoV, which caused the global respiratory infectious disease pandemic (worldwide), have been detected in the oral cavity of patients diagnosed with novel coronavirus. Studies have shown that [25] angiotensin converting enzyme 2 (ACE2), the main host cell receptor of coronavirus, is highly expressed in oral mucosa, especially the tongue epithelial cells. This also explains why high viral loads in saliva and throat swab samples from the vast majority of infected individuals are feasible for detecting novel coronavirus. Therefore, Huang et al. (2021) investigated the oral viral load in COVID-19 infected patients and concluded that the oral cavity may be one of the important routes for SARS-CoV-2 transmission [26]. A study by Da Silva Santos et al. revealed that the use of mouthwash, in addition to standard care, reduced viral load in the oral cavity, thereby reducing the length of hospital stay [27]. The Centers for Disease Control and Prevention (CDC) has advocated the benefits of preprocedural mouthwashes in reducing airborne pathogens of all types before clinical procedures [28]. Studies by Bernardo da Fonseca Orcina [29] and Marcelo Lupion Poleti et al. [30] indicated that mouthwashes containing antimicrobial phthalocyanine derivative (APD) had been found to have a positive impact on the relief of early clinical symptoms of patients with COVID-19, such as sore throat, cough, and mouth ulcers.

Prior to this, Cavalcante-Leao BL et al. included the results of two in vitro trials in a review that attempted to verify the efficacy of mouthwashes in reducing viral load. The results showed that the mouthwash containing PVP-I solution with a concentration of 1% (without dilution) and one of 7% (diluted at 1:30) examined in this systematic review had a killing effect on bacteria and viruses [31]. A review by Hernandez-Vasquez A et al., according to the present systematic review, indicated that the effect of mouthwash on SARS-CoV-2 viral load in the saliva of COVID-19 patients remained uncertain. Evidence from well-designed RCTs is required for further and more objective evaluation of this effect [32]. Majdy Idrees et al. [33]performed a meta-analysis of in vitro and in vivo experiments about the effect of mouthwash and nasal spray on viral load reduction, respectively, and concluded that a variety of active ingredients in mouthwash have confirmed therapeutic effects on SARS-CoV-2, but the duration of action of each active ingredient in vivo was not clear.

Therefore, questions remained regarding the use of mouthwash in SARS-CoV-2 patients before receiving clinical treatment.

Most studies have adopted CT values and viral loads to assess the posttreatment efficacy of COVID-19. In response to the SARS-CoV-2 pandemic, the CDC 2019 novel Coronavirus (2019-nCoV) Real-Time PCR Diagnostic Panel was approved by U.S. Food and Drug Administration and adopted by the NIH Clinical Center, herein referred to as the SARS-CoV-2 RT–PCR assay. Coronaviruses have a number of molecular targets within their positive-sense, single-stranded RNA genome that can be used for PCR assays. These include genes encoding structural proteins, including envelope glycoproteins spike (S), envelope (E), transmembrane (M), helicase (Hel), and nucleocapsid (N). In addition to the genes that encode structural proteins, there are species-specific accessory genes that are required for viral replication, including RNA-dependent RNA polymerase (RdRp), hemagglutinin-esterase (HE), and open reading frame 1a (ORF1a) and ORF1b [34]. Although SARS-CoV-2 RT-PCR is the gold standard for viral load estimation, this assay is semi-quantitative. Therefore, some studies have used reverse transcription-polymerase chain reaction (RT-PCR) to quantify viral load. Virus copies were normalized by mL of saliva. In this regard, it should be noted that viral loads of over 106 copies/ml were required for infectivity studies [35]. In view of the difficulties in culturing SARS-CoV-2 virus from clinical specimens, the current use of viral RNA load as a substitute remains reasonable [36].

This meta-analysis, which combined the results of 13 RCTs, provided strong evidence for the effectiveness of mouthwash in reducing novel coronavirus load. In the current study, CT value and viral load were the outcome measures for analyzing the effect of mouthwash on novel coronavirus. According to subgroup analysis by measurement time, it was concluded that the use of mouthwash was effective within 30 min in reducing viral load compared with routine oral care with placebo. According to subgroup analysis of the mouthwash ingredients, PVP-I-containing mouthwashes significantly elevated the CT value, which was consistent with the results of existing in vitro experimental findings, and SARS-CoV-2 virus could be completely inactivated with PVP-I-containing oral disinfectant in vitro [37]. PVP-I is composed of iodine and the water-soluble polymer polyvinylpyrrolidone. PVP-I has antimicrobial activity when it dissociates and releases iodine. The action of mouth rinses containing PVP-I against SARS-CoV-2 is due to the sensitivity of the virus to oxidation [38]. CHX is a cationic surfactant and synthetic biguanide with broad-spectrum antimicrobial activity, and is widely used as an antiseptic formulation in dental practice [39]. The results of this study showed that CHX-containing mouthwash was second only to PVP-I-containing mouthwash in reducing viral load. According to studies by Y Hanna Huang [40] et al., chlorhexidine was highly effective in preventing SARS-CoV-2 infection for some medical workers, with no infections among medical workers during the use of chlorhexidine, while the prevalence of novel coronavirus in medical workers from general hospitals approached 50% during the same period. Besides, Matheus Dos Santos Fernandez et al. systematically reviewed the killing effect of CHX on some virus strains, and the results showed that CHX had a good inactivation effect on herpes simplex virus-1 and influenza virus A. However, CHX is less effective in the elimination of influenza virus A compared with povidone-iodine [41]. HP is a broad-spectrum antibacterial agent, and is especially effective against coronavirus and influenza viruses [42]. This was also confirmed in a subgroup analysis of CT values presented in this review. CPC, BAC, β-cyclodextrin and citrox, as widely used antimicrobials, did not show statistically significant differences in the subgroup analysis of decreasing viral load of COVID-19, and more clinical trials are needed to demonstrate this finding.

In terms of safety, no adverse reactions were mentioned in the included 13 articles, and no relevant systematic reviews have reported any increase in the risk of oral disease caused by mouthwash. However, it should be noted that PVP-I is contraindicated in patients with thyroid disease, who are allergic to iodine and radioiodine therapy, or during pregnancy [43].

Compared with previous systematic reviews, this study is a meta-analysis based entirely on in vivo experiments. While discussing the effects of different ingredients of mouthwash on SARS-CoV-2 viral load, we also explored the effects of different mouthwash duration on SARS-CoV-2 viral load. This provides a positive reference for the clinical prevention of SARS-CoV-2. However, this study has some limitations in that some of the included studies had short and various follow-up durations, and high-quality studies with longer follow-up durations are still needed. Therefore, any conclusions from pooled outcome measures and their interpretations should be treated with caution. More RCTs of large-scale, high-quality, and large-sample may be needed in the future to validate the efficacy of various mouthwash ingredients in relieving SARS-CoV-2 symptoms after infection.

## Conclusions

In summary, mouthwash has some efficacy in reducing the viral load of novel coronavirus, especially within 30 min after rinsing the mouth. Mouthwash containing CHX, PVP-I and HP all had significant positive effects on CT values, and PVP-I-containing mouthwash may be a promising option to control novel coronavirus infections and relieve virus-related symptoms. However, studies on the dose and frequency of use of mouthwash for infection control are still lacking, which may limit the clinical application of mouthwash.

### Supplementary Information

**Additional file 1: Figure S1.** Sensitivity analysis of CT values.

**Additional file 2: Figure S2.** Sensitivity analysis of viral loads.

**Additional file 3: Figure S3.** Funnel plot of CT values.

**Additional file 4: Figure S4.** Funnel plot of viral loads.

**Additional file 5: Figure S5.** Filled funnel plot of CT values.

**Additional file 6: Table S1.** Search strategy.

## Data Availability

All data generated or analyzed during this study are included in this published article (and its Supplementary Information files).
