# Supplementary figures and images for: Efficacy of mouthwash on reducing salivary SARS-CoV-2 viral load and clinical symptoms: a systematic review and meta-analysis
Source: BMC Infect Dis. 2023 Oct 11;23:678. doi: 10.1186/s12879-023-08669-z (PMC10568889; doi:10.1186/s12879-023-08669-z)

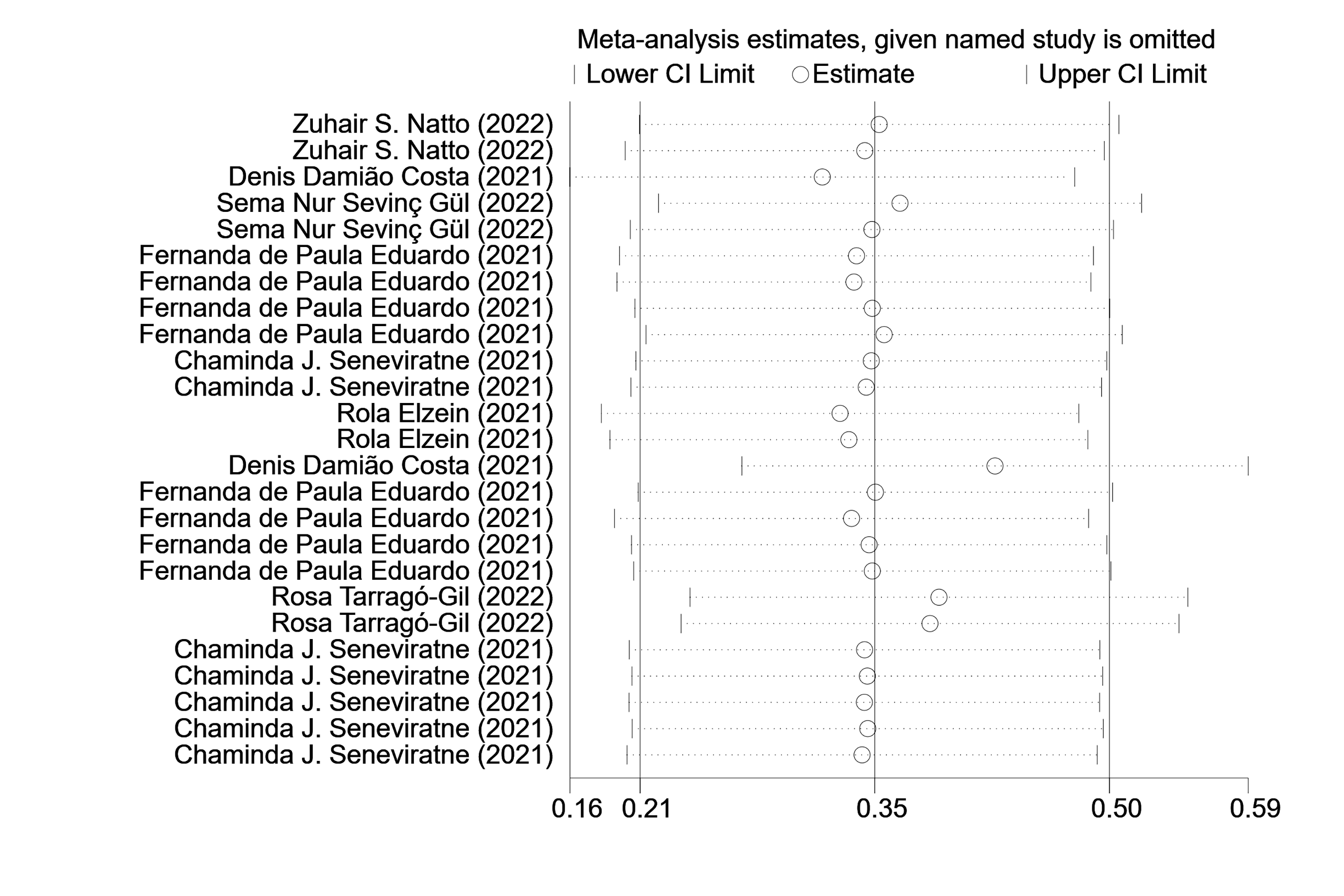

Supplement: Supplementary file 1 — Additional file 1: Figure S1. Sensitivity analysis of CT values. [file 12879_2023_8669_MOESM1_ESM.tif]

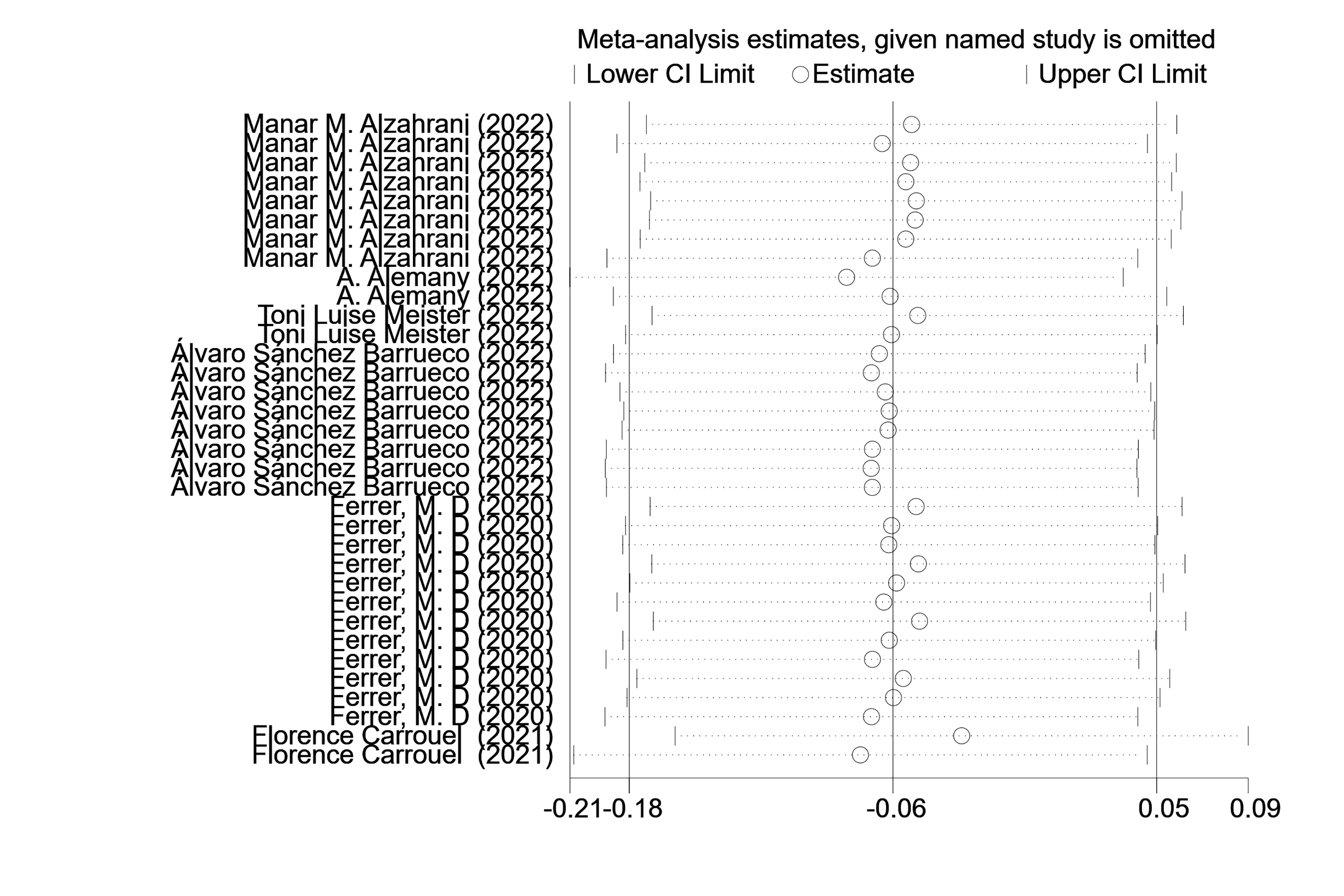

Supplement: Supplementary file 2 — Additional file 2: Figure S2. Sensitivity analysis of viral loads. [file 12879_2023_8669_MOESM2_ESM.tif]

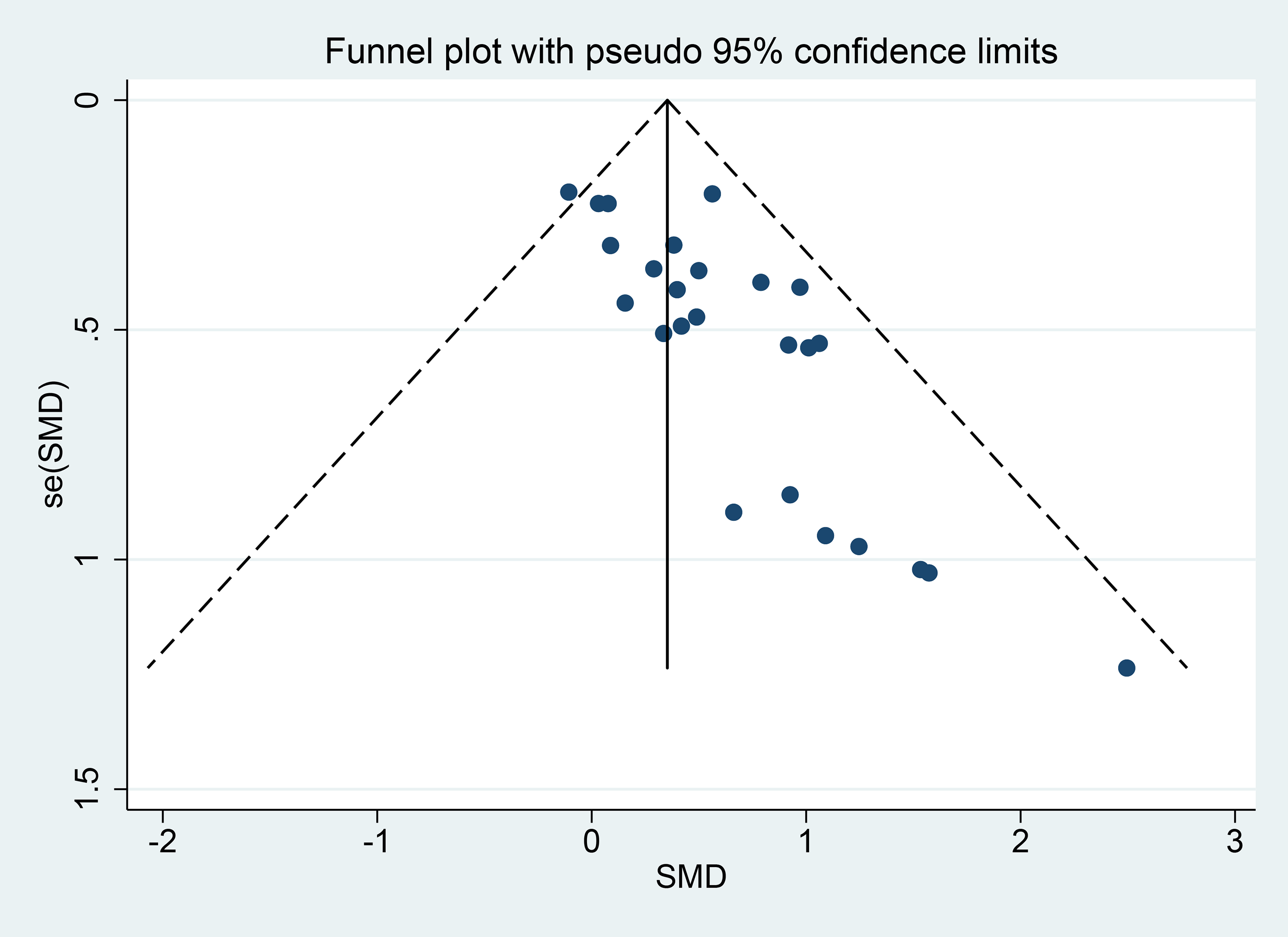

Supplement: Supplementary file 3 — Additional file 3: Figure S3. Funnel plot of CT values. [file 12879_2023_8669_MOESM3_ESM.tif]

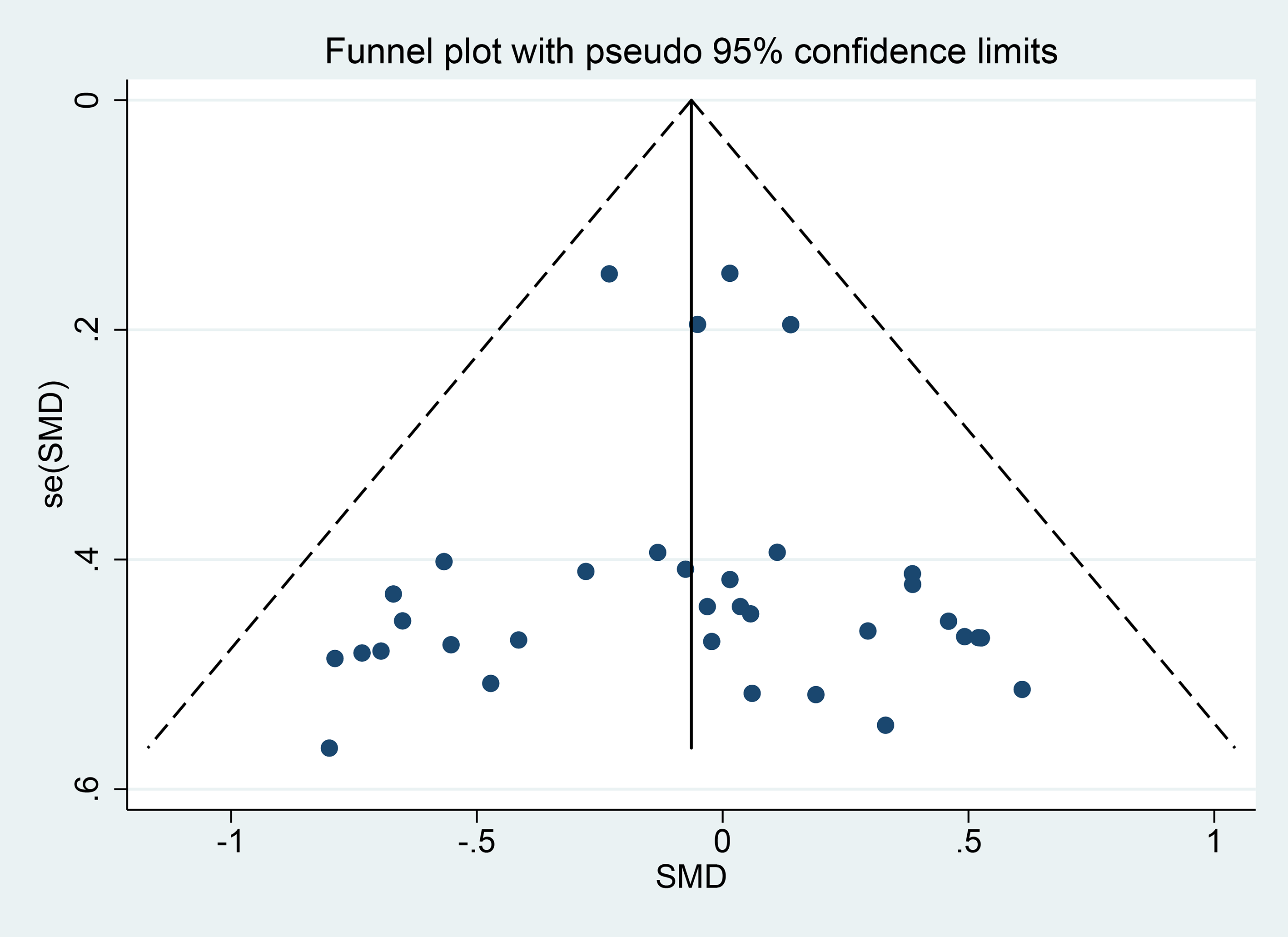

Supplement: Supplementary file 4 — Additional file 4: Figure S4. Funnel plot of viral loads. [file 12879_2023_8669_MOESM4_ESM.tif]

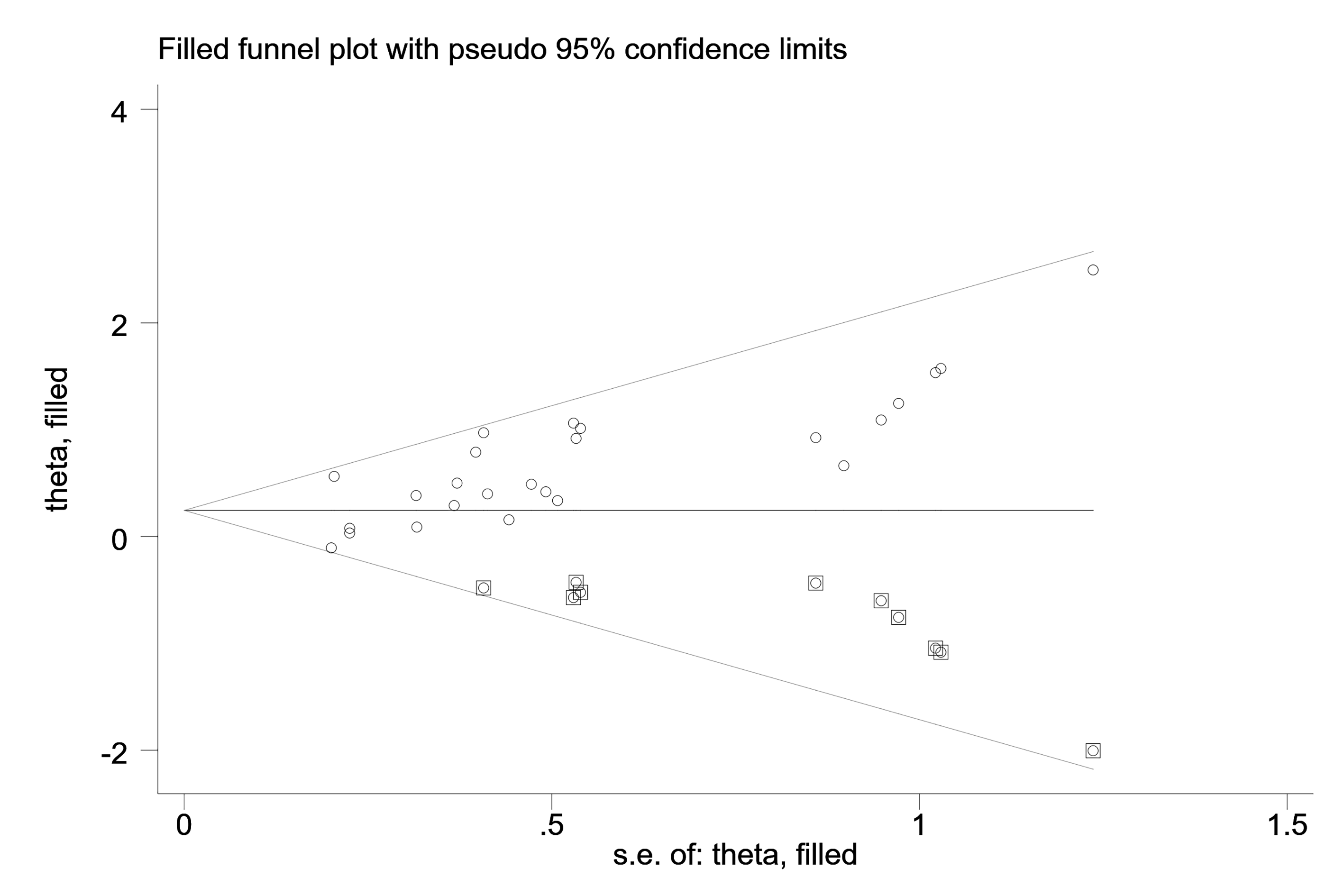

Supplement: Supplementary file 5 — Additional file 5: Figure S5. Filled funnel plot of CT values. [file 12879_2023_8669_MOESM5_ESM.tif]
