# Supplementary material for: Efficacy of mouthwash on reducing salivary SARS-CoV-2 viral load and clinical symptoms: a systematic review and meta-analysis
Source: BMC Infect Dis. 2023 Oct 11;23:678. doi: 10.1186/s12879-023-08669-z (PMC10568889; doi:10.1186/s12879-023-08669-z)
Supplement: Supplementary file 6 — Additional file 6: Table S1. Search strategy. [file 12879_2023_8669_MOESM6_ESM.docx]

**Table S1 Search strategy and registered protocol**

| **Search strategy** | |
| --- | --- |
| **PubMed** | For **PubMed**, the search used was:  (((((Mouthwashes[MeSH Terms]) OR (mouthrinse*[Title/Abstract])) OR (Mouth Bath*[Title/Abstract])) OR (MouthWash[Title/Abstract])) OR (Colgate Plax Overnight[Title/Abstract])) AND (((((((((((((((((((((((((((((((((((((((COVID-19[MeSH Terms]) OR (SARS-CoV-2[MeSH Terms])) OR (2019 nCoV[Title/Abstract])) OR (2019 new coronavirus[Title/Abstract])) OR (2019 novel coronavirus[Title/Abstract])) OR (2019 severe acute respiratory syndrome coronavirus 2[Title/Abstract])) OR (Coronavirus Disease 19[Title/Abstract])) OR (coronavirus disease 2[Title/Abstract])) OR (coronavirus infection 2019[Title/Abstract])) OR (coronavirus SARS-2[Title/Abstract])) OR (COVID[Title/Abstract])) OR (HCoV-19[Title/Abstract])) OR (Human coronavirus 2019[Title/Abstract])) OR (nCoV 2019 disease[Title/Abstract])) OR (nCoV 2019 infection[Title/Abstract])) OR (nCoV-2019[Title/Abstract])) OR (new coronavirus pneumonia[Title/Abstract])) OR (novel 2019 coronavirus[Title/Abstract])) OR (novel coronavirus 2019[Title/Abstract])) OR (novel coronavirus-19[Title/Abstract])) OR (SARS Coronavirus 2[Title/Abstract])) OR (SARS CoV 2[Title/Abstract])) OR (SARS2 (virus[Title/Abstract]))) OR (SARS-2-CoV[Title/Abstract])) OR (SARS-related coronavirus 2[Title/Abstract])) OR (Sever acute respiratory syndrome coronavirus 2[Title/Abstract])) OR (Severe acute respiratory coronavirus 2[Title/Abstract])) OR (Severe acute respiratory syndorme coronavirus 2[Title/Abstract])) OR (Severe acute respiratory syndrome 2 coronavirus[Title/Abstract])) OR (severe acute respiratory syndrome 2 virus[Title/Abstract])) OR (Severe Acute Respiratory Syndrome Coronavirus 2[Title/Abstract])) OR (Severe acute respiratory syndrome coronoavirus 2[Title/Abstract])) OR (Severe acute respiratory syndrome coronvirus 2[Title/Abstract])) OR (severe acute respiratory syndrome CoV-2 virus[Title/Abstract])) OR (Severe acute respiratory syndrome related coronavirus 2[Title/Abstract])) OR (Severe acute respiratory syndrome virus 2[Title/Abstract])) OR (Severe acute respiratoy syndrome coronavirus 2[Title/Abstract])) OR (Wuhan coronavirus[Title/Abstract])) OR (Wuhan seafood market pneumonia virus[Title/Abstract])) |
| **EMBASE** | For **EMBASE**, the search used was: ('mouthwash'/exp OR mouthrinse*:ti,ab,kw OR 'mouth bath*':ti,ab,kw OR 'MouthWash':ti,ab,kw OR 'colgate plax overnight':ti,ab,kw) AND ('coronavirus disease 2019'/exp OR 'severe acute respiratory syndrome coronavirus 2'/exp OR ('2019 ncov':ti,ab,kw OR '2019 new coronavirus':ti,ab,kw OR '2019 novel coronavirus':ti,ab,kw OR '2019 severe acute respiratory syndrome coronavirus 2':ti,ab,kw OR 'coronavirus disease 19':ti,ab,kw OR 'coronavirus disease 2':ti,ab,kw OR 'coronavirus infection 2019':ti,ab,kw OR 'coronavirus sars-2':ti,ab,kw OR covid:ti,ab,kw OR 'hcov 19':ti,ab,kw OR 'human coronavirus 2019':ti,ab,kw OR 'ncov 2019 disease':ti,ab,kw OR 'ncov 2019 infection':ti,ab,kw OR 'ncov 2019':ti,ab,kw OR 'new coronavirus pneumonia':ti,ab,kw OR 'novel 2019 coronavirus':ti,ab,kw OR 'novel coronavirus 2019':ti,ab,kw OR 'novel coronavirus-19':ti,ab,kw OR 'sars coronavirus 2':ti,ab,kw OR 'sars cov 2':ti,ab,kw OR (sars2:ti,ab,kw AND virus:ti,ab,kw) OR 'sars 2 cov':ti,ab,kw OR 'sars-related coronavirus 2':ti,ab,kw OR 'sever acute respiratory syndrome coronavirus 2':ti,ab,kw OR 'severe acute respiratory coronavirus 2':ti,ab,kw OR 'severe acute respiratory syndorme coronavirus 2':ti,ab,kw OR 'severe acute respiratory syndrome 2 coronavirus':ti,ab,kw OR 'severe acute respiratory syndrome 2 virus':ti,ab,kw OR 'severe acute respiratory syndrome coronavirus 2':ti,ab,kw OR 'severe acute respiratory syndrome coronoavirus 2':ti,ab,kw OR 'severe acute respiratory syndrome coronvirus 2':ti,ab,kw OR 'severe acute respiratory syndrome cov-2 virus':ti,ab,kw OR 'severe acute respiratory syndrome related coronavirus 2':ti,ab,kw OR 'severe acute respiratory syndrome virus 2':ti,ab,kw OR 'severe acute respiratoy syndrome coronavirus 2':ti,ab,kw OR 'wuhan coronavirus':ti,ab,kw OR 'wuhan seafood market pneumonia virus':ti,ab,kw)) |
| **Cochrane library** | For **Cochrane library**, the search used was:  #1 MeSH descriptor: [Mouthwashes] explode all trees  #2 (mouthrinse*):ti,ab,kw OR (Mouth Bath*):ti,ab,kw OR (MouthWash):ti,ab,kw OR (Colgate Plax Overnight):ti,ab,kw  #3 MeSH descriptor: [COVID-19] explode all trees  #4 MeSH descriptor: [SARS-CoV-2] explode all trees  #5 (2019 nCoV):ti,ab,kw OR (2019 new coronavirus):ti,ab,kw OR (2019 novel coronavirus):ti,ab,kw OR (2019 severe acute respiratory syndrome coronavirus 2):ti,ab,kw OR (Coronavirus Disease 19):ti,ab,kw OR (HCoV-19):ti,ab,kw OR (coronavirus disease 2):ti,ab,kw OR (coronavirus infection 2019):ti,ab,kw OR (coronavirus SARS-2):ti,ab,kw OR (COVID):ti,ab,kw OR (Human coronavirus 2019):ti,ab,kw OR (nCoV 2019 disease):ti,ab,kw OR (nCoV 2019 infection):ti,ab,kw OR (nCoV-2019):ti,ab,kw OR (new coronavirus pneumonia):ti,ab,kw OR (novel 2019 coronavirus):ti,ab,kw OR (novel coronavirus 2019):ti,ab,kw OR (novel coronavirus-19):ti,ab,kw OR (SARS Coronavirus 2):ti,ab,kw OR (SARS CoV 2):ti,ab,kw OR (SARS-related coronavirus 2):ti,ab,kw OR (Sever acute respiratory syndrome coronavirus 2):ti,ab,kw OR (Severe acute respiratory coronavirus 2):ti,ab,kw OR (Severe acute respiratory syndorme coronavirus 2):ti,ab,kw OR (Severe acute respiratory syndrome 2 coronavirus):ti,ab,kw OR (severe acute respiratory syndrome 2 virus):ti,ab,kw OR (Severe Acute Respiratory Syndrome Coronavirus 2):ti,ab,kw OR (Severe acute respiratory syndrome coronoavirus 2):ti,ab,kw OR (Severe acute respiratory syndrome coronvirus 2):ti,ab,kw OR (severe acute respiratory syndrome CoV-2 virus):ti,ab,kw OR (Severe acute respiratory syndrome related coronavirus 2):ti,ab,kw OR (Severe acute respiratory syndrome virus 2):ti,ab,kw OR (Severe acute respiratoy syndrome coronavirus 2):ti,ab,kw OR (Wuhan coronavirus):ti,ab,kw OR (Wuhan seafood market pneumonia virus):ti,ab,kw  #6 #1 OR #2  #7 #3 OR #4 OR #5  #8 #6 AND #7 |
| **Web of Science** | For **Web of Science**, the search used was:  #1 ((((TS=(Mouthwashes)) OR TS=(mouthrinse*)) OR TS=(Mouth Bath*)) OR TS=(Mouth Wash)) OR TS=(Colgate Plax Overnight)  #2 ((((((((((((((((((((((((((((((((((((((TS=(COVID-19)) OR TS=(SARS-CoV-2)) OR TS=(2019 nCoV)) OR TS=(2019 new coronavirus)) OR TS=(2019 novel coronavirus)) OR TS=(2019 severe acute respiratory syndrome coronavirus 2)) OR TS=(Coronavirus Disease 19)) OR TS=(coronavirus disease 2)) OR TS=(coronavirus infection 2019)) OR TS=(coronavirus SARS-2)) OR TS=(COVID)) OR TS=(HCoV-19)) OR TS=(Human coronavirus 2019)) OR TS=(nCoV 2019 disease)) OR TS=(nCoV 2019 infection)) OR TS=(nCoV-2019)) OR TS=(new coronavirus pneumonia)) OR TS=(novel 2019 coronavirus)) OR TS=(novel coronavirus 2019)) OR TS=(novel coronavirus-19)) OR TS=(SARS Coronavirus 2)) OR TS=(SARS CoV 2)) OR TS=(SARS2 (virus))) OR TS=(SARS-2-CoV)) OR TS=(SARS-related coronavirus 2)) OR TS=(Sever acute respiratory syndrome coronavirus 2)) OR TS=(Severe acute respiratory coronavirus 2)) OR TS=(Severe acute respiratory syndorme coronavirus 2)) OR TS=(Severe acute respiratory syndrome 2 coronavirus)) OR TS=(severe acute respiratory syndrome 2 virus)) OR TS=(Severe Acute Respiratory Syndrome Coronavirus 2)) OR TS=(Severe acute respiratory syndrome coronoavirus 2)) OR TS=(Severe acute respiratory syndrome coronvirus 2)) OR TS=(severe acute respiratory syndrome CoV-2 virus)) OR TS=(Severe acute respiratory syndrome related coronavirus 2)) OR TS=(Severe acute respiratory syndrome virus 2)) OR TS=(Severe acute respiratoy syndrome coronavirus 2)) OR TS=(Wuhan coronavirus)) OR TS=(Wuhan seafood market pneumonia virus)  #3 #1 AND #2 |
| **Registered protocol** | |
| The protocol was registered at *https://www.crd.york.ac.uk/prospero/display_record.php?ID=CRD* | |
